# CNV Radar: an improved method for somatic copy number alteration characterization in oncology

**DOI:** 10.1186/s12859-020-3397-x

**Published:** 2020-03-06

**Authors:** David Soong, Jeran Stratford, Herve Avet-Loiseau, Nizar Bahlis, Faith Davies, Angela Dispenzieri, A. Kate Sasser, Jordan M. Schecter, Ming Qi, Chad Brown, Wendell Jones, Jonathan J. Keats, Daniel Auclair, Christopher Chiu, Jason Powers, Michael Schaffer

**Affiliations:** 1 Janssen Research & Development, LLC, 1400 McKean Road, Spring House, PA 19477 USA; 2 Q2 Solutions, EA Genomics, Morrisville, NC USA; 3 Unite de Genomique du Myelome, Institut Universitaire du Cancer de Toulouse-Oncopole, Toulouse, France; 4 University of Calgary, Arnie Charbonneau Cancer Institute, Calgary, AB Canada; 5 Myeloma Institute, Little Rock, AR USA; 6 Mayo Clinic, Division of Hematology, Rochester, MN USA; 7 Genmab, Princeton, NJ USA; 8 Janssen Research & Development, LLC, Raritan, NJ USA; 9 OmicSoft Corporation, Cary, NC USA; 10 Translational Genomics Research Institute, Phoenix, AZ USA; 11 Multiple Myeloma Research Foundation, Norwalk, CT USA

**Keywords:** Copy number variation, Next generation sequencing, Multiple myeloma, Acute myeloid leukemia, Prostate cancer

## Abstract

**Background:**

Cancer associated copy number variation (CNV) events provide important information for identifying patient subgroups and suggesting treatment strategies. Technical and logistical issues, however, make it challenging to accurately detect abnormal copy number events in a cost-effective manner in clinical studies.

**Results:**

Here we present CNV Radar, a software tool that utilizes next-generation sequencing read depth information and variant allele frequency patterns, to infer the true copy number status of genes and genomic regions from whole exome sequencing data. Evaluation of CNV Radar in a public multiple myeloma dataset demonstrated that CNV Radar was able to detect a variety of CNVs associated with risk of progression, and we observed > 70% concordance with fluorescence in situ hybridization (FISH) results. Compared to other CNV callers, CNV Radar showed high sensitivity and specificity. Similar results were observed when comparing CNV Radar calls to single nucleotide polymorphism array results from acute myeloid leukemia and prostate cancer datasets available on TCGA. More importantly, CNV Radar demonstrated its utility in the clinical trial setting: in POLLUX and CASTOR, two phase 3 studies in patients with relapsed or refractory multiple myeloma, we observed a high concordance rate with FISH for del17p, a risk defining CNV event (88% in POLLUX and 90% in CASTOR), therefore allowing for efficacy assessments in clinically relevant disease subgroups. Our case studies also showed that CNV Radar is capable of detecting abnormalities such as copy-neutral loss of heterozygosity that elude other approaches.

**Conclusions:**

We demonstrated that CNV Radar is more sensitive than other CNV detection methods, accurately detects clinically important cytogenetic events, and allows for further interrogation of novel disease biology. Overall, CNV Radar exhibited high concordance with standard methods such as FISH, and its success in the POLLUX and CASTOR clinical trials demonstrated its potential utility for informing clinical and therapeutic decisions.

## Background

Copy number alterations or variations (CNAs or CNVs) play an important role in human disease and biology [1]. For example, germline CNAs are associated with large scale alterations, such as trisomy 21, 18, and 13 which cause Down’s, Edwards’, and Patau’s syndrome, respectively [2], autism [3, 4], and other severe birth defects [5]. Somatic CNAs (SCNAs), on the other hand, are commonly observed in cancer and are major drivers for tumor development and drug resistance [6, 7]. Such SCNA events occur both at the gene and the chromosome levels: pan-cancer genomic analyses have reported frequent amplification of *MYC* and deletion of *PTEN* and *TP53* in many tumor types [6]. In acute myeloid leukemia (AML), deletions involving large portions of chromosomes 5 and 7 are frequently seen in patients with unfavorable cytogenetic risk [8]. In multiple myeloma, deletion of chromosome 17 is associated with more aggressive disease and acquisition of chromosome 17 deletion during disease progression confers a worse prognosis [9]. Moreover, deletion of *TP53* or amplification of chromosome 1 leads to deregulation of genes involved in myeloma pathogenesis (e.g. *CKS1B*, *MCL1*) and is associated with poor prognosis [10–13]. In contrast, presence of hyperdiploidy (concurrent gains of multiple chromosomes such as 3, 5, 7, 9, 11, 15, 19 and 21) [14] is associated with favorable outcomes with extended patient response after high-dose melphalan-based therapies and other therapies [13, 15–17]. Characterization of cancer-associated copy number events is therefore valuable for identifying patient subgroups and provides insights into prognosis and potential treatment strategies.

The detection of SCNAs in cancer samples has traditionally been performed by cytogenetic and microarray-based technologies such as fluorescent in situ hybridization (FISH), array comparative genomic hybridization (CGH), and Affymetrix single nucleotide polymorphism array 6.0 (SNP6 array) [18]. While FISH has been widely used in clinical applications for detection of specific abnormalities [19], it is limited by the number of loci that it can simultaneously investigate and also by the availability of FISH probes for pre-specified regions of interest. Next-generation sequencing (NGS) is becoming increasingly popular for studying genomic variations in cancer. Whole genome sequencing (WGS) allows for genome-wide detection of CNAs, translocations, and breakpoints. However, in the clinical setting, a capture-based approach that interrogates the exome (whole exome sequencing; WES) or a panel of cancer genes in a cost-effective manner can be preferred [20].

Several bioinformatics methods exist to call CNAs from WGS data [21–24]. Additionally, due to popularity of WES in clinical sequencing, several methods have been developed for copy number analysis of WES data [25], including ExomeDepth [26], copy number inference from exome reads (CoNIFER) [27], CopywriteR [28], and CNVkit [29]. ExomeDepth compares reads mapped to a region of interest in the test sample with reads mapped in the reference set using a beta-binomial model to control for technical variability at library preparation, capture and sequencing [26]. CoNIFER, on the other hand, attempts to detect and remove technical biases from a study cohort using singular value decomposition (SVD) [27]. To handle the large variation in capture efficiency of targeted capture regions, CopywriteR excludes all reads mapping to capture regions and uses only off-target reads to infer CNAs [28]. CNVkit takes an augmented approach at estimating CNAs in samples by utilizing both the targeted regions and the non-specifically captured off-target reads to infer copy number more evenly across the genome [29].

These algorithms primarily use relative read depths to derive the copy number status of the sample of interest. However, read depth alone is not sufficient to provide information critical for interpreting cancer genomes, such as copy-neutral loss of heterozygosity (CN-LOH), tumor-normal admixture, and potential sample contamination. In the clinical oncology setting, typically there are limited matched normal samples collected or sequenced during clinical studies due to budget restriction or sample availability, further posing challenges to algorithms that require paired normals. Utilizing a panel of unmatched normal samples is an alternative approach for somatic CNV detection recommended by CNVkit [29] and GATK4 [30].

Here, we present CNV Radar (CNV Rapid aberration detection and reporting), a new CNV calling algorithm that addresses challenges such as lack of matched controls and technical biases due to bait sizes, location, and hybridization conditions, by utilizing a panel of normal samples sequenced in similar conditions to the tumor sample. In addition to read depth information at regions of interest, CNV Radar’s statistical model uses variant allele frequency (VAF; also known as B-Allele Frequency [31]) patterns to infer the copy number status. VAF is the proportion of aligned reads at a common single nucleotide polymorphism (SNP) location that carry the alternate allele; therefore, in a normal diploid sample, each heterozygous locus has an expected VAF of 0.5, whereas the deletion or amplification of a chromosome produces different expected VAFs (e.g. a single copy gain leads to VAFs shifting towards 1/3 or 2/3). Since multiple germline SNPs typically coexist in a copy number altered region, their VAF information when used together as a group provides more signal for detecting CNVs than individual mutations. This information not only facilitates the estimation of copy numbers but also allows for the identification of CN-LOH and hyperdiploidy events that are commonly observed in cancer. A comparison of the advantages and disadvantages of read depth- versus VAF-based approaches is outlined in Table 1.

**Table 1:** Advantages and disadvantages of using relative read depths vs. VAF for determining CNV

CNV detection approach	Advantages	Disadvantages
Read depth	1. Uses any locus with reasonable coverage, providing higher resolution. 2. Dependent on proper mapping, not on variant calls. 3. Relatively robust to small contamination events from another human sample.	1. Highly susceptible to technical bias both from batch to batch and from sample to sample due to variable sensitivity of the various regions to moderate or even slight changes in hybridization conditions and other factors (such as exome enrichment kit versions). When used by itself, this approach can yield a large number of false positives and negatives if not sufficiently validated. 2. Unable to identify complex copy number events such as CN-LOH or hyperdiploidy.
Variant allele frequency (VAF)	1. Uses heterozygote positions to discern bands that indicate larger copy number changes. 2. More resistant to technical bias from batch to batch and from sample to sample.	1. Can confuse copy number events with contamination events. 2. Cannot discern concurrent gains in both alleles from normal state. 3. Misinterprets CN-LOH events as copy number loss events. 4. Requires that variant calling and heterozygous states be established. Resolution depends on the number and distribution of variants called.
Read depth combined with VAF	1. One method fills in the gaps in information from the other method. 2. Properly assesses CN-LOH events and concurrent gains in both alleles as well as copy number state when small levels of contamination occur.	1. Inherently different resolution levels complicate creation of individual segments from both sources of information.

*VAF* variant allele frequency; *CNV* copy number variation; *CN-LOH* copy-neutral loss of heterozygosity

To illustrate the performance of CNV Radar in real-world scenarios, we evaluated it on several large-scale cancer datasets including multiple myeloma, AML, and prostate cancer, and compared the exome-derived CNVs with CNVs defined by FISH, WGS, and microarray assays. We also compared its performance to several other CNV callers, and evaluated it in samples collected from two global phase 3 studies in patients with relapsed or refractory multiple myeloma [32, 33]. Overall, CNV Radar is accurate and sensitive across these datasets, provides genomic information important for interpreting tumor samples (e.g. identification of risk biomarkers and interrogation of emerging biology), and can potentially guide treatment strategies for patients.

## Results

### CNV Radar for CNV and CN-LOH detection

**Fig. 1:**
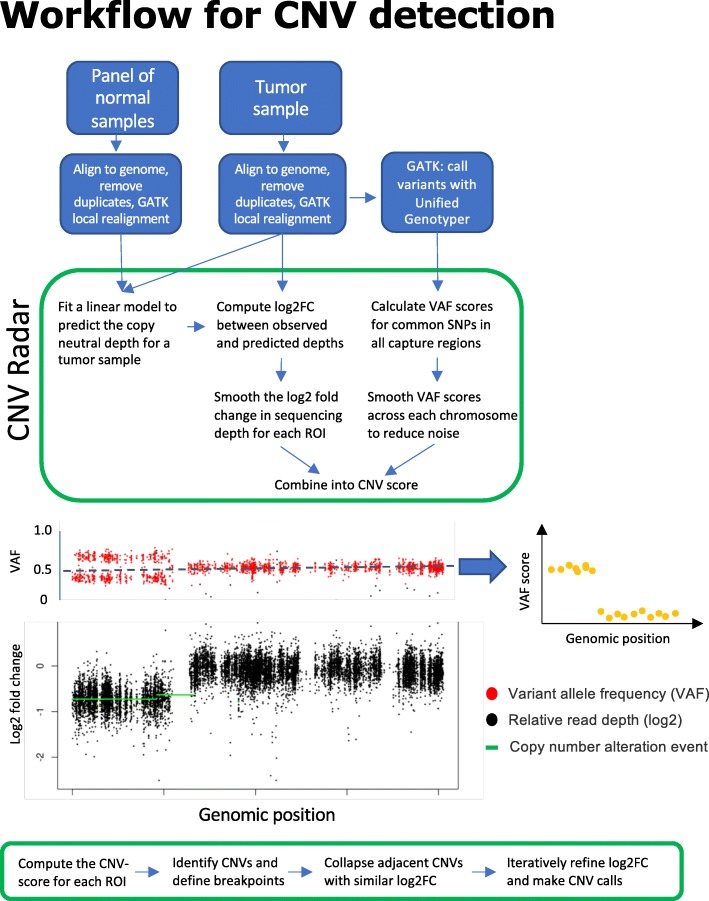
CNV detection workflow. The tumor and normal samples were first aligned to the human genome and processed independently to remove PCR duplicates and perform local realignment. Common SNPs were detected in the tumor sample using GATK [30]. CNV Radar then uses the alignment files from tumor and normal samples to calculate relative read depths (log2 fold change; black) at all capture regions. VAF scores (orange), which indicate the level of deviation from a copy neutral state, were calculated for all heterozygous common SNPs in the tumor sample. The smoothed log2 fold change and VAF scores were then combined into CNV scores, which CNV Radar then uses to iteratively detect breakpoints, re-center the genome-wide read depth, and make the final CNV calls (green). CNV, copy number variation; GATK, Genome Analysis Tool Kit; VAF, variant allele frequency

To evaluate the performance of CNV Radar, we first analyzed the WES data from a subset of patient samples from the Multiple Myeloma Research Foundation (MMRF) CoMMpass study (https://www.themmrf.org), which is a landmark initiative in the field of multiple myeloma research with the goal of mapping 1000 patients’ genomic profiles to clinical outcomes and enabling development of a more complete understanding of patients’ responses to treatments (**Methods and materials**). This subset of patients also have CNV information derived from matched WGS or FISH assays. Following the CNV detection workflow (Fig. 1), all tumor and normal WES samples were independently aligned and pre-processed for SNP detection and read depth calculation. For each capture region, we calculated the average (mean) read depth across all of its sequenced bases. The dendrogram of the normal samples clustered by the average read depth at all capture regions showed heterogeneity possibly due to varying library preparation conditions, exome capture efficiencies, or sample quality (Additional file 1). As no obvious outliers were observed, all normal samples were used as references to determine CNVs.

**Fig. 2:**
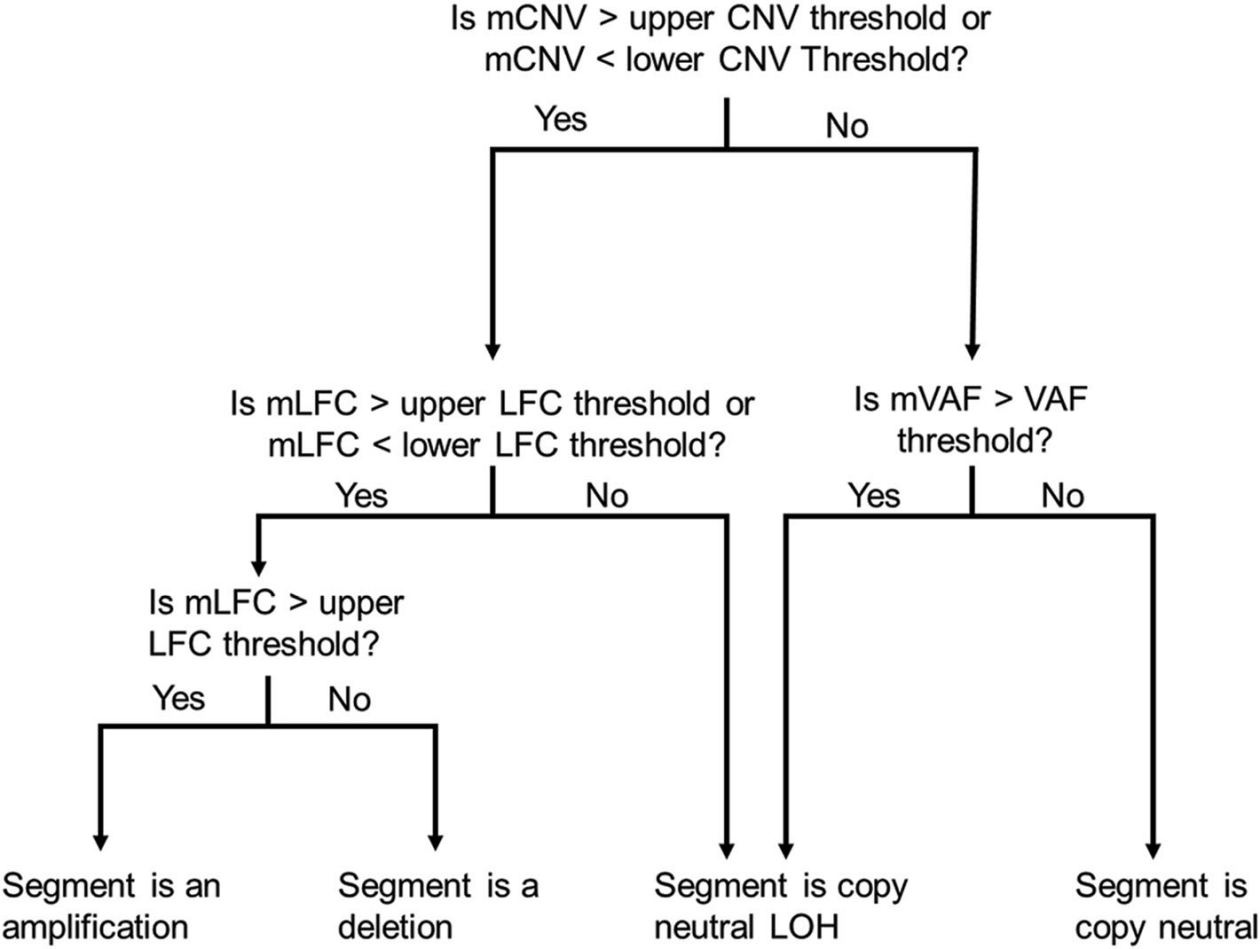
Schematic diagram for assigning copy number types to each genomic segment that was defined by identified breakpoints. mCNV, median copy number variation; CNV, copy number variation; mLFC, median log_2_ fold change; LFC, log_2_ fold change; mVAF, median variant allele frequency; VAF, variant allele frequency; LOH, loss of heterozygosity

**Fig. 3:**
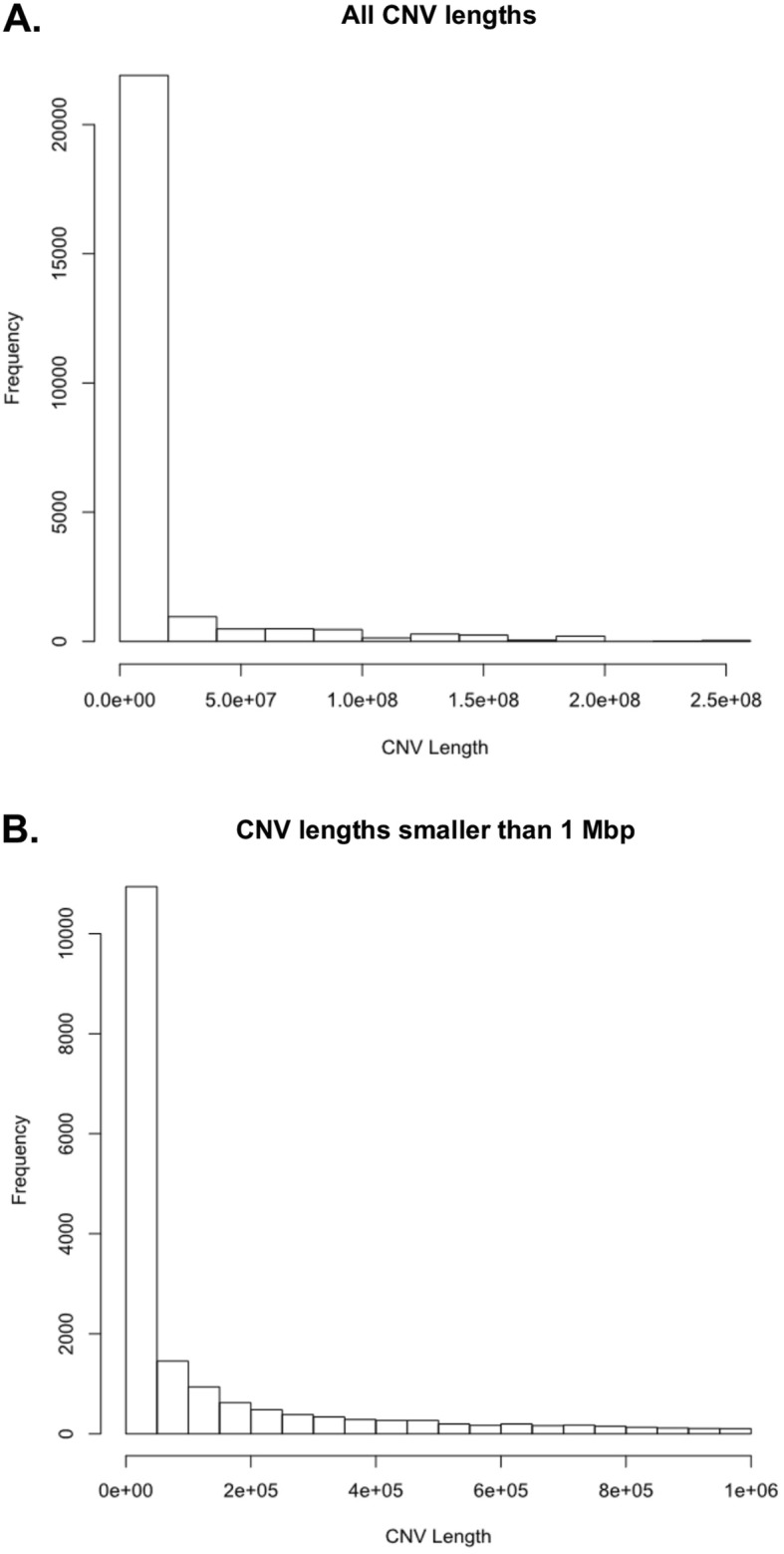
CNV Radar detects CNV events at varying scales. (**a**) Distribution of CNV events at all lengths. (**b**) Distribution of CNV events smaller than 1 Mbp. CNV Radar, copy number variation rapid aberration detection and reporting

**Fig. 4:**
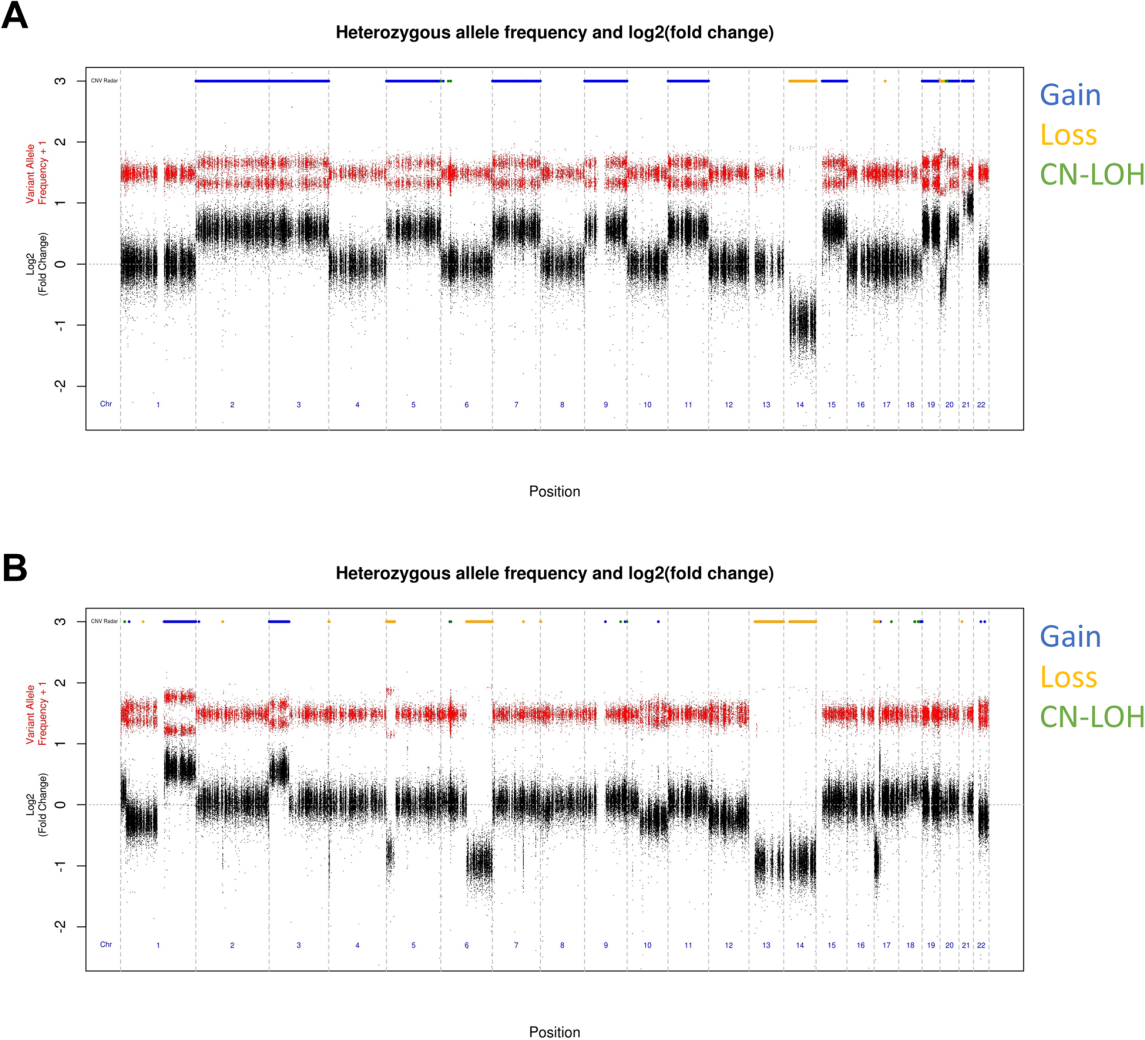
Genome-wide plots of relative read depth and VAF for (**a**) a hyperdiploidy example (SRR2128547) (**b**) a sample (SRR2128541) that had risk associated cytogenetic changes (amp1q, del13, and del17p). Top panel of horizontal bars indicates calls by CNV Radar. Red dots indicate 1 + VAF. Black dots indicate relative read depth measured by the log_2_ ratio of normalized tumor depth vs. normalized reference depth. VAF, variant allele frequency; CNV Radar, copy number variation Rapid aberration detection and reporting, CN-LOH, copy-neutral loss of heterozygosity

CNV Radar normalizes the read depths, estimates the relative copy ratio using a regression model, and through an iterative process over 3 rounds calculates a VAF score that indicates deviation from a copy neutral state. The relative read depth and VAF score are then combined to detect breakpoints in the genome and make CNV calls (Fig. 2; **Methods and materials**). Across the tumor samples, CNV Radar detected an average of 233 CNV events, with a median length of 109,800 bp (Fig. 3). Closer inspection of the copy number events showed focal and large-scale CNAs consistent with myeloma biology such as deletion of *TP53*, monosomy, hyperdiploidy, and deletion of chromosomes 13 and 17p (Fig. 4). As chromosomes 1p, 1q, 13, and 17p are known regions associated with multiple myeloma risk and commonly measured in patients by FISH, we further examined CNV Radar calls at the four marker regions. Since these large scale events typically span most of the chromosome arm (e.g. chr1q), the marker level CNV status was determined based on the detection of CNV in ≥ 50% of the region by CNV Radar. Comparison of CNV Radar calls with FISH results showed > 70% concordance (Table 2). While in a small number of cases CNV Radar had missed CNV calls due to complex rearrangement events, most discordant cases were attributed to tumor heterogeneity or low number of tumor cells harboring the CNV.

**Table 2:** Concordance between CNV Radar calls and FISH results

	TP	FP	FN	TN	Total	Concordance
**del1p**	6	3	4	96	109	93.58%
**amp1q**	23	1	29	56	109	72.48%
**del13q14**	45	4	12	48	109	85.32%
**del17p13**	6	1	11	91	109	88.99%

*CNV Radar* copy number variation Rapid aberration detection and reporting; *FISH* fluorescence in situ hybridization; *TP* true positive; *FP* false positive; *FN* false negative; *TN* true negative

**Fig. 5:**
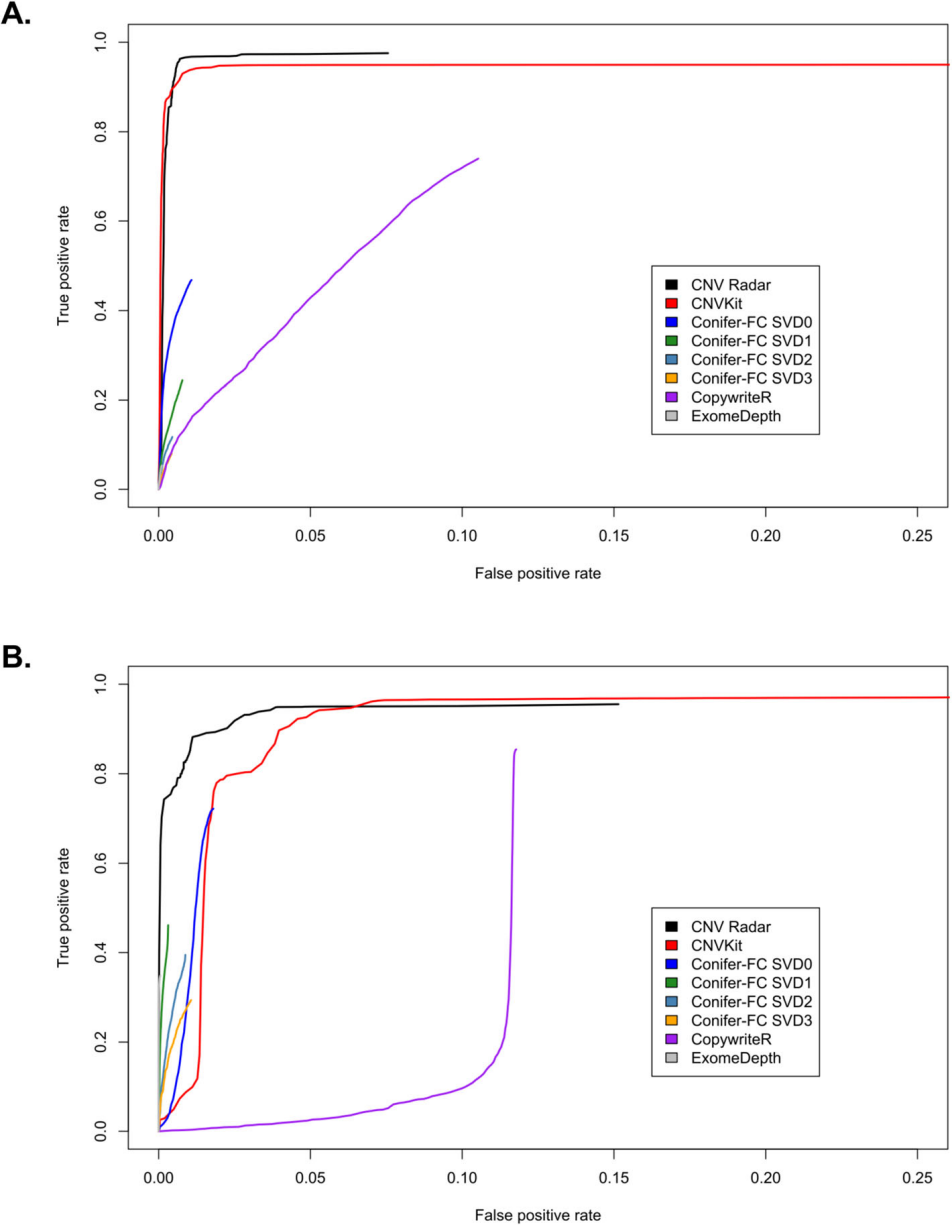
Comparison of the calls made by CNV Radar to those defined by WGS on the MMRF CoMMpass dataset. (**a**) ROC curve for amplification detection. (**b**) ROC curve for deletion detection. CNV Radar, copy number variation rapid aberration detection and reporting; WGS, whole genome sequencing; MMRF, Multiple Myeloma Research Foundation; ROC, receiver operating characteristic

We also evaluated the sensitivity and specificity of CNV detection on WES data by comparing to CNVs defined by WGS (as reported by MMRF), and evaluated the performance of four other commonly used CNV callers: CNVkit, CoNIFER, CopywriteR, and ExomeDepth (see Additional file 2). It is worth mentioning that two methods, PennCNV2 and ASCAT [34, 35], which were originally developed for CNV detection from SNP arrays, also utilize VAF information; therefore, in principle these methods could be adapted to analyze WES data. However, as the WES versions of these methods are still under development and do not allow the use of pooled normal references, they were excluded from this evaluation. In our performance evaluation, ground truth CNVs were defined by WGS because, in contrast to WES, WGS provides full sequencing coverage of the entire genome and is not affected by exome capture biases. CNV Radar showed high sensitivity and specificity as demonstrated by the receiver operating characteristic (ROC) curves and area under the ROC curve (AUC) values (Fig. 5, Table 3**; Methods and materials**). The performance of CNV Radar was also comparable or superior to other commonly used CNV callers (Table 3), with CNV Radar and CNVkit showing the highest AUC values.

**Table 3:** AUC of the evaluated CNV callers on the MMRF CoMMpass WGS dataset

CNV Caller	AUC (gain)	AUC (loss)
CNV Radar	0.94	0.90
CNVkit	0.93	0.65
CoNIFER – FCSVD0	0.45	0.59
CoNIFER – FCSVD1	0.23	0.45
CoNIFER – FCSVD2	0.11	0.37
CoNIFER – FCSVD3	0.08	0.28
CopywriteR	0.25	0.01
ExomeDepth	0.05	0.35

*AUC* area under the receiver operating characteristic curve; *CNV* copy number variation; *MMRF* Multiple Myeloma Research Foundation; *WGS* whole genome sequencing; *Radar* rapid aberration detection and reporting; *CoNIFER* copy number inference from exome reads

We further evaluated the sensitivity of CNV detection at the sample level for the two best performing algorithms: CNV Radar and CNVkit. Across the 109 multiple myeloma patient samples evaluated, CNVkit had a median sensitivity of 95.8%, while CNV Radar had a median sensitivity of 97.3% (Additional file 3). Median positive predictive values (which equals 1-false discovery rate), defined as the total number of true positive (TP) calls divided by the sum of the TP and false positive (FP) calls were 98.0% for CNVkit, and 98.1% for CNV Radar. Given the comparable performance between the two algorithms, we manually characterized cases where CNV Radar and CNVkit performed most differently.

**Fig. 6:**
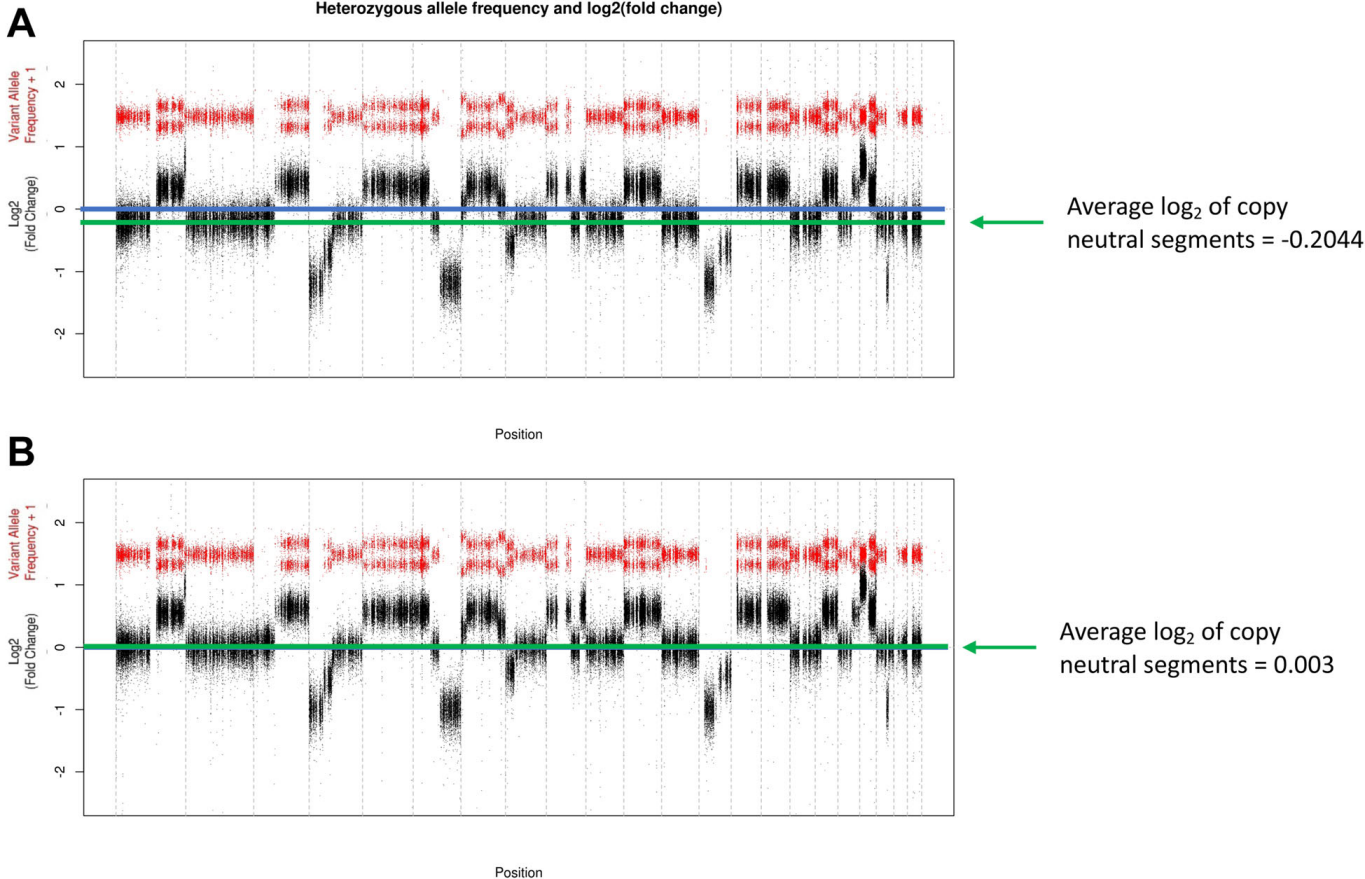
A myeloma tumor (SRR2128492) with complex CNAs. (**a**) Initial LFC across the genome. (**b**) Corrected LFC across the genome. The average log_2_ ratio of copy neutral segments changed from − 0.2044 in the first iteration (**a**) to 0.003 in the last (**b**). Horizontal blue lines indicate a copy ratio of 0. CNA, copy number alteration; LFC, log2 fold change

As CNV Radar utilizes information from both read depth and VAF, it is particularly suited for inferring the CNV status in massively altered genomes such as myeloma where read depth based methods typically have trouble correctly normalizing and establishing the baseline. For example, in sample SRR2128492, the relative read depth between the tumor and normal samples showed a biased genome-wide background possibly due to complex copy number alterations and differences in the exome enrichment efficiency (Fig. 6a). It was, therefore, challenging to infer the correct copy number status using read depth information alone: the unadjusted log_2_ fold change (LFC) values suggested large-scale amplification of more than a dozen regions and the deletion of the rest of the entire genome. CNV Radar iteratively used VAF to identify copy neutral regions and re-establish the baseline LFC. As a result, the LFC was correctly estimated and CNVs could be accurately detected (Fig. 6b). On the contrary, CNVkit was unable to properly re-center the baseline LFC and infer the correct CNV status of this sample. Overall, the genome-wide level of copy number changes had a slight impact on the performance of both CNV callers: in cases where CNV Radar was more sensitive than CNVkit, a median of 923 M bases were modified as defined by WGS truth, whereas samples where CNVkit performed better had a median of 370 M modified bases.

### Evaluation of CNV Radar on the AML and prostate TCGA datasets

**Fig. 7:**
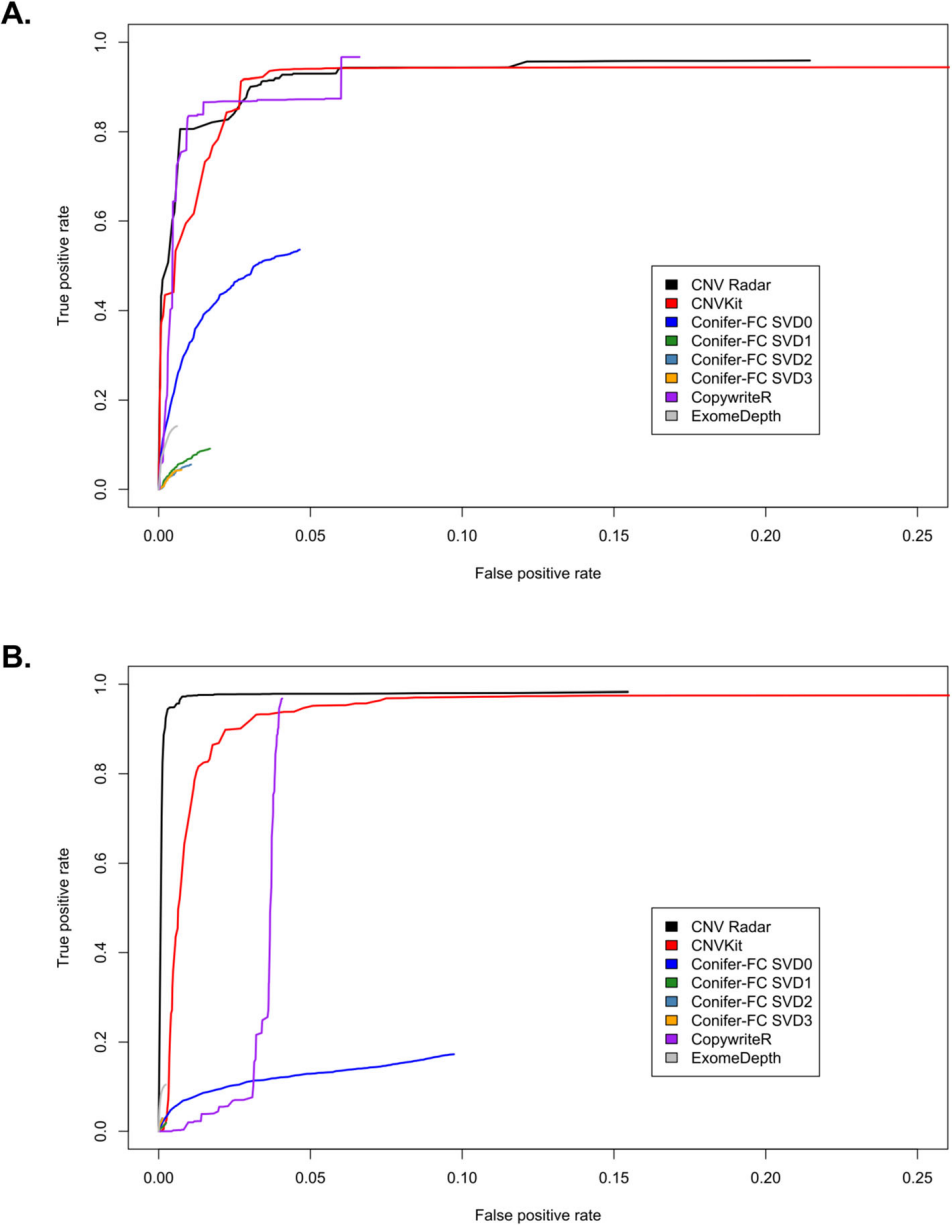
Comparisons of the calls made by CNV Radar to results from the Affymetrix SNP6 arrays for the TCGA AML dataset (200 patients). (**a**) ROC curves for amplification detection. (**b**) ROC curves for deletion detection. CNV Radar, copy number variation rapid aberration detection and reporting; SNP, single nucleotide polymorphism; TCGA, The Cancer Genome Atlas; AML, acute myeloid leukemia; ROC, receiver operating characteristic

**Fig. 8:**
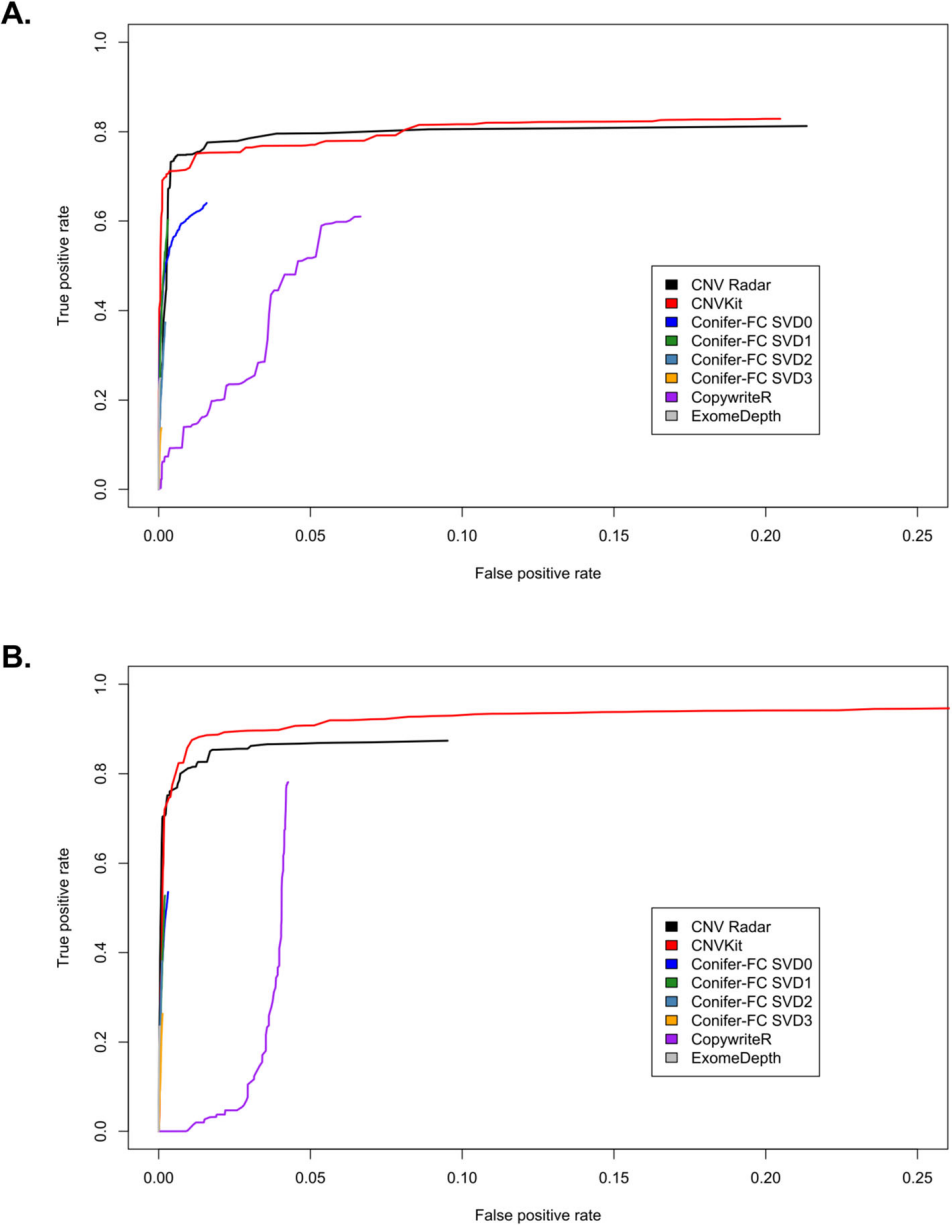
Comparison of the calls made by CNV Radar to results from the Affymetrix SNP6 arrays for the TCGA prostate dataset (333 patients). (**a**) ROC curves for amplification detection. (**b**) ROC curves for deletion detection. CNV Radar, copy number variation rapid aberration detection and reporting; SNP, single-nucleotide polymorphism; TCGA, The Cancer Genome Atlas; ROC, receiver operating characteristic

In addition to the MMRF CoMMpass study, we examined the performance of CNV Radar on two TCGA datasets (AML and prostate) [8, 36] and compared the CNV Radar calls to CNVs derived from SNP6 arrays in each study. We also used these datasets to evaluate the performance of several other CNV callers: CNVkit, CoNIFER, CopywriteR, and ExomeDepth. In both TCGA AML (Fig. 7, Table 4) and prostate (Fig. 8, Table 5) datasets, the ROC curves and AUCs showed that CNV Radar and CNVkit had comparable results and had the best performance among the evaluated CNV callers.

**Table 4:** AUC of the evaluated CNV callers on the TCGA AML dataset

Caller	AUC (gain)	AUC (loss)
CNV Radar	0.83	0.96
CNVkit	0.79	0.80
CoNIFER_FCSVD0	0.43	0.10
CoNIFER_FCSVD1	0.08	0.02
CoNIFER_FCSVD2	0.05	0.03
CoNIFER_FCSVD3	0.04	0.03
CopywriteR	0.79	0.29
ExomeDepth	0.14	0.10

*AUC* area under the receiver operating characteristic curve; *CNV* copy number variation; *TCGA* The Cancer Genome Atlas; *AML* acute myeloid leukemia; *Radar* Rapid aberration detection and reporting; *CoNIFER* copy number inference from exome reads

**Table 5:** AUC of the evaluated CNV callers on the TCGA prostate dataset

Caller	AUC (gain)	AUC (loss)
CNV Radar	0.75	0.83
CNVkit	0.75	0.86
CoNIFER_FCSVD0	0.62	0.53
CoNIFER_FCSVD1	0.59	0.52
CoNIFER_FCSVD2	0.37	0.38
CoNIFER_FCSVD3	0.14	0.26
CopywriteR	0.26	0.21
ExomeDepth	0.25	0.24

*AUC* area under the receiver operating characteristic curve; *CNV* copy number variation; *TCGA* The Cancer Genome Atlas; *Radar* rapid aberration detection and reporting; *CoNIFER* copy number inference from exome reads

### Application of CNV Radar in a clinical trial setting

The efficacy of daratumumab, an anti-CD38 monoclonal antibody, in combination with lenalidomide and dexamethasone or bortezomib and dexamethasone was evaluated in patients with relapsed or refractory multiple myeloma in POLLUX and CASTOR, respectively [32, 33]. High-risk cytogenetics is a key prognostic factor in multiple myeloma and to determine if daratumumab would be efficacious in high risk patients (defined as having at least one of t [4;14], t [14;16], or del17p cytogenetic abnormalities), bone marrow aspirates were collected from 311 patients from POLLUX and 353 patients from CASTOR at screening. Exome-seq was performed and copy number status was evaluated by CNV Radar, and independent experts manually reviewed the sequencing depth and VAF patterns to verify the status of amp1q, del13, and del17p; 98.6% of the CNV Radar calls were confirmed by experts. A subset of these patients was also evaluated by FISH, and the concordance rate for del17p between these two methodologies was 88% in POLLUX and 90% in CASTOR. Lower rates of concordance were observed for del13 (70 and 64%, respectively) and amp1q (72 and 70%, respectively), which may be due to variations in how FISH data was entered at local sites (e.g. number of positive cells or number of chromosome copies required to define a case), and the possibility that some CNAs identified by CNV Radar were not captured by FISH [37].

**Fig. 9:**
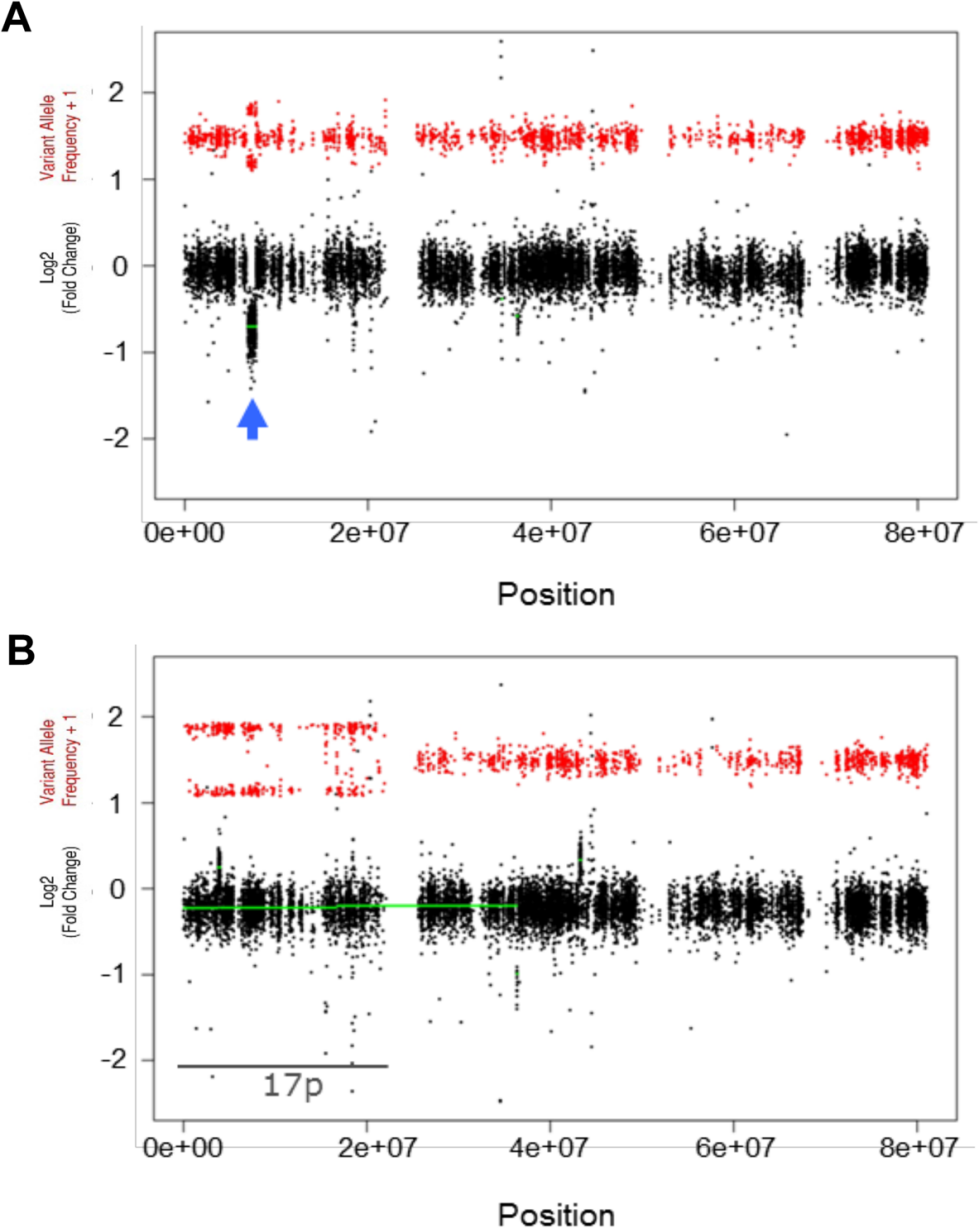
Leveraging CNV Radar to uncover additional risk factors. (**a**) Focal deletion in TP53 detected by CNV Radar. Arrow indicates region that is deleted. (**b**) Copy-neutral loss of heterozygosity in 17p. CNV Radar, copy number variation rapid aberration detection and reporting

Given the complexity of the myeloma genomes and the prognostic value of copy number changes [13, 38], the utility of CNV Radar in investigating disease biology is highlighted by case studies from these phase 3 trials. In one patient who was determined to be negative for del17p by both exome-sequencing and FISH, CNV Radar detected a focal deletion in *TP53* that may represent a functional deletion of this chromosomal region (Fig. 9a). In a different patient who was also determined to be del17p-negative by exome-sequencing (no FISH results were reported), alteration of the VAF without loss of relative read depth measured by CNV Radar revealed CN-LOH of the 17p region, where the loss of a copy of 17p and amplification of the remaining copy shifted the VAF bands towards 0 and 1 (Fig. 9b). These examples highlight the value of exome-sequencing in detecting clinically important cytogenetic events not detectable by conventional FISH.

## Discussion

Although NGS has been routinely applied in medical genetics [39], the application in oncology has primarily focused on the detection of mutations, and gains or losses in a limited number of cancer genes [40]. With increased cost-efficiency, WES-based tests are now being used in a clinical oncology setting and allow for global detection of mutations and copy number changes in coding regions of the genome, although more sensitive and accurate methodologies for defining copy number changes are needed [41].

We developed CNV Radar to overcome the technical and logistical hurdles that prevent accurate detection of CNVs in clinical samples. Although WES typically captures less than 2% the genome and relies on probes and PCR amplification that can introduce library prep-specific biases, we demonstrated that CNV Radar’s performance was comparable to other genomics methods for copy number identification. Using samples from the MMRF CoMMpass study, CNV Radar achieved 99.5% sensitivity when compared to WGS data, suggesting that WGS is not required for accurate detection of most CNV events. In addition, when compared to FISH calls, we obtained an average concordance rate of 85%. CNVs detected by SNP6 arrays in TCGA AML and prostate cancer samples also agreed with CNV Radar calls.

Although FISH and SNP6 arrays have been considered gold standard methods for CNV detection, there remain concerns with both technologies. Compared to newer genomics technologies, FISH has poor resolution and it is therefore difficult to detect small events and precise breakpoints. Furthermore, it has relatively low throughput and requires time-consuming manual curation, and can only detect specific abnormalities of pre-determined interest. Interlaboratory variability also makes it challenging to interpret FISH data derived from different laboratories [42]. Similarly for SNP6 arrays, Pinto et al. reported < 50% concordance of various SNP calling algorithms on the same raw SNP array data and < 70% concordance of calls from the same platform and algorithmic software, but used replicate preparations for almost all SNP array platforms [43]. Pinto et al. further reported a concordance rate of 80% between replicates for one lab but approximately 60% between replicates for another lab [43].

Although CNV Radar utilizes both read depth and VAF information for CNV detection, it still occasionally requires manual tuning by the user. For example, the MMRF sample that had the most false negative (FN) bases by CNV Radar compared to CNVkit was SRR2128693, where CNV Radar marginally missed the CNV threshold for the entire chromosomes 2, 3, and 4 (Additional file 4). Also, large regions of double amplifications, if present in the majority of the cells, would cause VAF to center around 0.5 and be missed by CNV Radar. In addition, although CNV Radar uses VAF information to detect CN-LOH, cross-sample contamination could lower the signal-to-noise ratio and reduce its performance. In this case, the user should evaluate the extent of the contamination. If most of the derived read depth from WES came from the tumor sample of interest, the user may adjust the parameters and have CNV Radar rely solely on read depth information (e.g. set *CNV*-*score* to $\hat{LFC}$, effectively discarding any VAF information). A related limitation is the inference of subclonal CNV events from bulk tumor sequencing data, which remains challenging to current copy number callers but can be addressed with advanced single cell technology. Lastly, although we demonstrated the detection of *TP53* deletions and detected small CNV events, the possible lack of heterozygous SNPs and low overlap with WES capture regions makes identification of focal alterations a challenging and active area of research. Future work to improve CNV Radar is ongoing to further incorporate different sources of information to accurately identify complex and focal events.

## Conclusion

The field of oncology drug development is growing increasingly dependent on the identification of biomarkers for drug approval [44–48]. We verified CNV Radar’s ability to accurately infer the copy number status of disease relevant biomarkers using TCGA AML and prostate samples, demonstrating its potential utility in both heme and solid cancers. We further applied CNV Radar to the CASTOR [32] and POLLUX [33] myeloma clinical trial datasets and found a strong concordance rate with FISH in identification of various myeloma risks associated CNA/CNV events such as amp1q, del13, and del17p. The application of CNV Radar to patient sample repositories, such as TCGA, and to future clinical trials could provide additional prognostic or predictive genomic characterization of patients and help accurately identify patients with high-cytogenetic risk to enable evaluation of therapeutic efficacy in this particularly vulnerable population.

## Methods

### Cancer samples

#### MMRF CoMMpass

We used the exome-seq data from the MMRF CoMMpass study [49] to assess the accuracy of CNV Radar and to demonstrate its application in the oncology setting. MMRF CoMMpass is an ongoing multi-institution collaboration that will create a comprehensive genomic database of 1000 multiple myeloma patients. Samples are collected at baseline and longitudinally for low-pass WGS [50], exome-seq, RNA-seq, and immunophenotyping. FISH is also performed to detect the copy number status for regions associated with myeloma risk (e.g. chromosomes 1q, 13, 17p). The MMRF selected a subset of 109 baseline patient samples and manually curated the FISH results to ensure the accuracy and consistency of data (e.g. done with plasma cell enrichment; FISH results properly transcribed into database). For the low-pass WGS data, copy number variants were identified by an analysis of differential coverage between each tumor and its matched normal sample. Relative copy number is determined as the log2 difference between the normal and tumor normalized coverage, where normalization is defined as the mean coverage across a 2 kb window divided by the genome-wide coverage. Circular binary segmentation (CBS) algorithm was used to segment copy number data. Allele frequency of common SNPs from matched tumor WES was further used to re-center the copy number data so regions with VAF of 0.5 became copy neutral. Segments with LFC values less than − 0.25 were defined as deleted, whereas segments with greater than 0.2 were defined as amplified. The scripts for generating the WGS CNV calls are available at https://github.com/tgen/MMRF_CoMMpass/tree/master/tCoNut_COMMPASS. In this manuscript, we defined the CNV status of patients based on MMRF-reported calls derived from WGS and FISH. To evaluate the performance and accuracy of different CNV callers, we used the exome data from these patients along with 141 normal samples (95 matched and 46 unmatched) to derive their copy number status and compared the results to those derived from the corresponding low-pass whole genome and FISH assays.

#### TCGA AML

TCGA analyzed a collection of genomes of 200 de novo AML patients [8]. Affymetrix SNP Array 6.0 was performed on both tumor and matched normal skin samples to derive copy number changes. Briefly, TCGA normalized intensity values using Partek Genomics Suite. Segmentation and copy number calling were done using CBS in the DNACopy package [51]. From the TCGA AML cohort, we selected 21 patients with known favorable risk and 39 patients with unfavorable risk, analyzed their WES data to make CNV calls with respective methods, and compared the results to the copy number status defined by the SNP6 array as reported in the paper [8]. With respect to the WES data, we downloaded the raw sequencing files from the Genomic Data Commons (GDC) Data Portal (https://portal.gdc.cancer.gov/), and used respective CNV calling pipelines to detect copy number changes.

#### TCGA prostate

TCGA performed molecular analysis of 333 primary prostate cancers to identify major subtypes among patients as well as potential treatment targets [36]. Tumor and matched normal specimens were characterized using platforms such as WES and Affymetrix SNP 6.0 arrays. We selected a subset of 30 patients who showed evidence of recurrent CNAs defined by the SNP6 array data and GISTIC2 [52, 53], as provided by Firehose (http://gdac.broadinstitute.org), and downloaded the raw WES data from GDC Data Portal to make CNV calls using respective CNV analysis pipelines. The results were compared to the ground truth defined by SNP6 arrays.

### Overview of CNV detection workflow

#### Mapping and pre-processing

The CNV Radar analysis pipeline (Fig. 1) starts with binary representations of sequence alignment map (BAM) files [54] of a tumor and a set of matched or un-matched normal samples that have been aligned, sorted, and indexed using standard alignment tools (e.g. Burrows-Wheeler Aligner (BWA) and SAMtools). Picard MarkDuplicates [55] is then used to remove PCR duplicates. The Genome Analysis Toolkit (GATK) [30] is used to perform local realignment of reads around indels and to identify single nucleotide variants (SNPs) in the tumor genome. SNP calls are annotated using The Single Nucleotide Polymorphism Database (dbSNP) [56] and snpEff [57, 58]. We utilize all heterozygous common SNPs for CNV detection since if a segment of the genome is amplified or deleted, all of the heterozygous common SNPs in the region will have altered VAF as a result. For example, in a homogeneous tumor sample, the VAFs will approach $\left[\frac{1}{\left(2+ CN\  gain\right)},\frac{\left(1+ CN\  gain\right)}{\left(2+ CN\  gain\right)}\right]$ in the case of copy number (CN) gain, and $\left[\frac{1- CN\  loss}{2- CN\  loss},\frac{1}{2- CN\  loss}\right]$ in the case of copy number loss. The analysis of the deviation of VAF from the copy neutral state as well as the pattern of this deviation in a group of neighboring SNPs in a genomic region of interest forms the basis of our method. The common SNPs are defined by dbSNP [56].

#### Read depth calculation and normalization

We define “read depth” as the number of times that a given nucleotide has been read in a WES experiment. For each capture region, we calculate the mean read depth across all of its sequenced bases. The mean read depths for each capture region, or capture depths*,* are calculated for each sample. Capture depths are then median scaled and log transformed across regions to account for differences in overall sequencing depth between samples.

#### Selection of normal references

Before CNV calls can be made, a normal baseline must be established. CNV Radar does not require matched tumor-normal samples. Instead, it establishes a normal depth baseline from a normal sample cohort. Due to the properties of the algorithmic implementation (linear regression, see below for more information), CNV Radar automatically up-weights those normal samples with a profile closest to the tumor sample allowing for good performance as long as at least a subset of the normal cohort has a depth profile similar to the tumor sample. However, it may still be beneficial to remove obvious outliers potentially caused by contamination or capture/sequencing failure. CNV Radar (available at the EA Genomics GitHub page [https://github.com/ExpressionAnalysis]) provides scripts that perform a clustering analysis of the set of normal samples to identify potential quality control issues. Using this cluster diagram, end users may remove outliers and select normal reference samples that best represent the sample population.

#### Estimation of log fold change by read depth

For each tumor sample, the copy number changes are estimated by comparing the tumor capture depths to depths from the normal references in log space. This is achieved by using a multiple linear regression model, which took the selected normal references as independent variables, and the transformed capture depth from the tumor sample as a dependent variable:

$$ {Y}_j\sim {\upbeta}_0+\sum \limits_{i=1}^n\left({\upbeta}_i{\mathrm{X}}_{ij}\right)+{\upvarepsilon}_i $$

In this model, Y_j_ is the log2 mean depth of capture region *j* in the tumor sample, X_ij_ is the log2 mean depth of sample *i* in the normal cohort for capture region *j*, and *n* is the number of samples in the normal cohort. β_0_ captures the bias that is present in both the tumor sample and the normal cohort (e.g. hybridization probe affinities) and each of the β_i_ captures the bias that is present in each sample (e.g. total read depth of the sample). We can then use the parameter for each sample to provide a weight for the observed mean depth for each of the capture regions found in the normal cohort to predict the expected normal copy number depth for the tumor. The expected normal copy number depth is therefore:

$$ \hat{Y_j}={\upbeta}_0+\sum \limits_{i=1}^n\left({\upbeta}_i{\mathrm{X}}_{ij}\right)+{\upvarepsilon}_i $$

And LFC for each capture region *j* is thus calculated as the difference between the log tumor read depth and the regression fitted values of log read depth using the normal references.

$$ {LFC}_j={Y}_j-\hat{Y_j} $$

This regression model implicitly gives higher weight to normal samples having more similarity in read depths to the current tumor sample. This reduces the impact of systematic variations that happen due to laboratory protocols or reagent lots, preservation techniques or tissue types. To reduce noise, we further calculated smoothed LFC values (${\hat{LFC}}_j$) for each capture region *j* using a smoothed spline of the LFCs across all capture regions.

#### Calculation of CNV breakpoints

Using the filtered VAFs from the GATK calls, we define a VAF score for every detected heterozygous position *i* in the genome:

$$ VAF-{score}_i={\left|{VAF}_i-0.5\right|}^3 $$

where *VAF*_*i*_ ∈ [0,1]. The exponent of 3 was set after empirical analysis using initial datasets. VAF-scores are then spline-smoothed and fitted values ($\hat{VAF-{score}_j}\Big)$ are made for each capture region *j*. For each chromosome, the $\hat{LFC_j}$ and $\hat{VAF-{score}_j}$ values were multiplied together to form *CNV* ­ *score*_*j*_ and call copy number events as follows:

$$ CNV-{score}_j=\min \left(\max \left({\hat{LFC}}_j,-3\right),3\right)\ast {\hat{VAF- score}}_j $$

The ceilings and thresholds for $\hat{LFC_j}$ ensure that the CNV-score is not completely dominated by the read-depth portion of the formula when analyzing read depth extremes. For single copy loss or gain in a typical admixture with normal cells, $\hat{LFC_j}$ may range from approximately [−0.4,0.3] while the $\hat{VAF-{score}_j}$ is naturally bound to [0,0.125] for any CN state. Next, the numerical derivative of the CNV-scores were calculated and used to identify breakpoints. Adjacent prospective CNVs having a large overlap in the interquartile ranges were merged into a single event. Specifically, if more than 20% of the $\hat{LFC}$ of the first CNV segment were contained within the interquartile range of the second CNV, or vice versa, then the two CNV segments were collapsed into a single CNV defined by their total region.

#### Determination of CNV state (loss, gain, or CN-LOH)

Genomic segments defined by the identified breakpoints were next categorized as loss, gain or CN-LOH. For each segment, median CNV-scores, $\hat{VAF}$ and $\hat{LFC}$ (mCNV, mVAF and mLFC, respectively) were calculated. Using separate thresholds for each metric, segments were classified as follows. A segment was classified as CN-LOH if its mCNV was within the upper and lower CNV thresholds, but the mVAF was above the VAF threshold. A segment was also classified as CN-LOH if mCNV was outside of the CNV thresholds, but its mLFC was within the LFC thresholds. Alternatively, a segment was classified as an amplification if its mCNV was outside of the CNV thresholds and the mLFC was above the upper LFC threshold. Finally, a segment was classified as a deletion if the mCNV was outside of the CNV thresholds, but the mLFC was below the lower LFC threshold. This categorization is illustrated in Fig. 2. The program defaults to threshold values which were established based on those values that provided the greatest accuracy (balancing FP and FN calls) on a training set of data. The truth for this reference data was determined through one or more orthogonal molecular assays and expert consensus manual review of detailed sequencing summaries (variants, variant frequencies, relative read depths) that were independent of any software CN algorithms. These default values should be appropriate in most scenarios, although parameters are available for tuning.

#### Iterative refinement of $\hat{LFC}$

Samples with CNAs in large portions of their genome can have biased estimates for $\hat{LFC}$ since these CNA regions contribute to a large proportion of the sequence reads and affect the sequencing coverage available for the rest of the genome. For example, given constant sequencing capacity, if half of the genome is amplified, the total reads from the amplified regions will increase substantially, leaving less room for reads from the rest of the normal genome and hence biasing $\hat{LFC}$ estimates downward, giving a false detection of deletion. For this reason, CNV Radar first calculates regions containing CNVs of highest absolute LFC. Excluding these biased regions, $\hat{LFC}$ is re-estimated for each capture region and CNVs are recalculated. This process is repeated three times (default) or for the number of iterations specified by the user.

### Performance evaluation and comparison with other CNV callers

#### Receiver operating characteristic (ROC) analysis

We assessed the performance of CNV callers by the ROC curve and AUC. ROC curves have the desirable property of visually displaying the trade-offs between sensitivity and specificity. However, they also require the existence of only two states, where CNAs have at least three: neutral, deletion and amplification. For this reason, TP, FP, true negatives (TNs) and FN calls were assigned according to Table 6 for deletions and amplifications. Here, each base was tallied separately, allowing for larger CNVs to have more weight in ROC analysis.

**Table 6:** Assignment of CNV caller results into TP, FP, TN, and FN as defined by FISH, WGS, or SNP arrays. Evaluation was performed separately for (A) deletions and (B) amplifications

A.		True State		
CNV		Neutral	Amplification	Deletion
State	Neutral	TN	TN	FN
Called	Amplification	TN	TN	FN
	Deletion	FP	FP	TP
B.		True State		
CNV		Neutral	Amplification	Deletion
State	Neutral	TN	FN	TN
Called	Amplification	FP	TP	FP
	Deletion	TN	FN	TN

*CNV* copy number variation; *TP* true positive; *FP* false positive; *TN* true negative; *FN* false negative; *FISH* fluorescence in situ hybridization; *WGS* whole genome sequencing; *SNP* single nucleotide polymorphism

The true positive rate (TPR) and the false positive rate (FPR) are defined separately for amplifications and deletions as follows:

$$ TPR=\frac{TP}{P}, FPR=\frac{FP}{N} $$

where TP is the number of bases with correctly inferred copy numbers, P the total number of bases with a copy number event defined by ground truth, FP the number of bases with incorrectly inferred copy numbers and N the total number of bases without a copy number event defined by ground truth, as defined in Table 6. The false positive rate is defined as the number of false positive bases divided by the sum of the false positive bases and the true negative bases. Calculating the number of true negative bases is done by subtracting the sum of the true positive, false positive, and false negative bases from the total bases in genome. For each CNV caller, outputs generated by each of the CNV calling tools were used, and we used varying thresholds on the corresponding CNV indicator variable to classify if a region had a copy number event. Generally, these tools only list the genomic coordinates identified as being part of a CNV. In these regions, the magnitude of the CNV (e.g. log2 fold change) and sometimes a metric of how much evidence exists for the CNV call are reported (e.g. Bayes factor for ExomeDepth). For this ROC analysis, copy number gain and loss were evaluated separately for each CNV caller, and the threshold is based on how much evidence/confidence each algorithm has that the base is part of a CNV call. When multiple metrics could be used to define ROC curves (e.g. *p*-value and fold change), the metric giving the best performance was chosen (i.e. LFC for CNVkit, CNV-score for CNV Radar, z-FPKM for CoNIFER, LFC for CopywriteR, and Bayes factor for ExomeDepth). As the thresholds were incremented on the confidence scores, regions absent from the output were not evaluable due to lack of confidence scores; therefore, the ROC curves for a particular caller may not go to 1 even by choosing an extreme threshold. As a result, an FPR of 1 was not able to be forced. Since the number of true negatives was generally much larger than the number of false positives, the portion of the ROC curve from 0 to 0.25 is displayed as this gives the best representation of the differences in the performance of the selected tool.

For the same reason, AUC was calculated as a proportion of the total area of the ROC curves defined by FPRs between 0 and 0.05. Thus, unlike the typical ROC analysis an AUC under 0.025 would mean that a random guess works better. Often, the total output of a caller, under the most liberal parameters and filtering, produced FPRs less than 0.05. In these cases, AUC was calculated by extending a horizontal line from the right-most endpoint of the ROC curve to 0.05. The ground truth CNV status is defined as segments with LFC values less than − 0.25 or greater than 0.2, thresholds that allow detection of single-copy CNVs in a tumor with purity as low as 30%.

#### Concordance rate

For multiple myeloma samples from the MMRF CoMMpass study, we evaluated the performance of CNV callers in chromosomal regions associated with myeloma risk: 1p, 1q, 13, and 17p. The CNV status of each region (marker level CNV) was first determined based on the detection of CNV in ≥ 50% of the region. The marker level CNV was then compared to FISH calls that were manually curated by the MMRF CoMMpass study team. The concordance rate was defined as the total number of subjects where the marker level CNV status by a CNV caller agrees with the ground truth set by FISH, divided by the total number of subjects.

For multiple myeloma samples from the phase 3 POLLUX and CASTOR studies [32, 33], chromosomal regions 1q, 13, and 17p were evaluated for CNVs using both CNV Radar (v1.0) and CNVkit by comparing changes in read depth to a normal reference that was generated using 100 peripheral blood mononuclear cell samples; 95.8% concordance was observed between the two callers. 100 discordant calls were manually reviewed by two independent experts in a central sequencing facility at Expression Analysis-Q^2^ Solutions (Morrisville, NC). Four cases too difficult to judge by human experts were called using CNV Radar (v1.1), which generated more detailed statistics across the regions of interest.

## Supplementary information

**Additional file 1.** Dendrogram of 141 normal samples based on clustering by read depth at all capture regions. Distance between samples was defined as 1 – Pearson correlation of read depth at capture regions.

**Additional file 2.** Supplementary material on running CNVkit, CoNIFER, ExomeDepth and CopywriteR.

**Additional file 3.** Sensitivity of CNV Radar (blue) and CNVkit (pink).

**Additional file 4.** Sample SRR2128693 was called more accurately by CNVkit than CNV Radar. Top panel of horizontal bars indicates CNV calls by CNV Radar and CNVkit as well as the true CNV status defined by WGS.

## Data Availability

CNV Radar is implemented in R and is available on the EA Genomics GitHub homepage [59]. The software is available for free under a non-commercial license (see the license on GitHub for more information).
