# Supplementary material for: CNV Radar: an improved method for somatic copy number alteration characterization in oncology
Source: BMC Bioinformatics. 2020 Mar 6;21:98. doi: 10.1186/s12859-020-3397-x (PMC7060549; doi:10.1186/s12859-020-3397-x)

Additional File 1

Dendrogram of 141 normal samples based on clustering by read depth at all capture regions. Distance between samples was defined as 1 – Pearson correlation of read depth at capture regions.

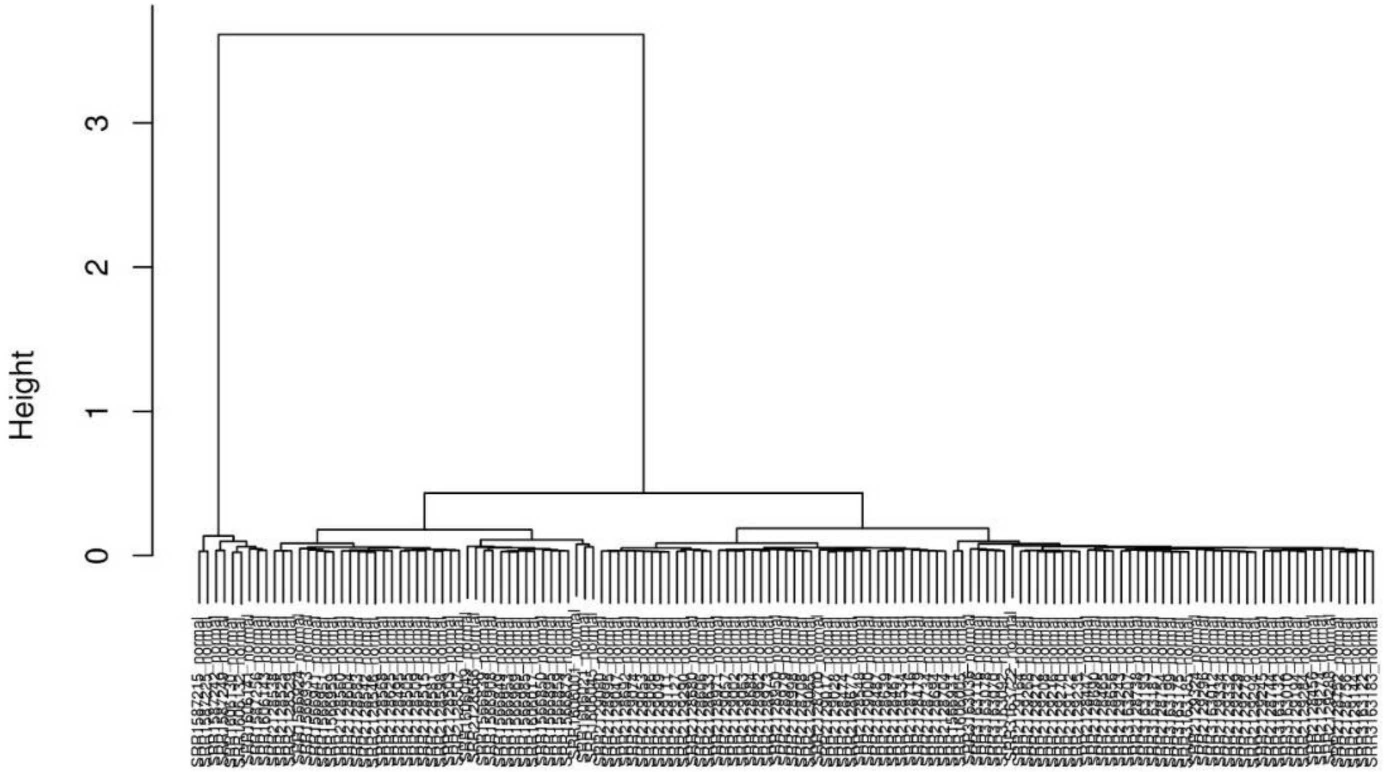

Supplement: Supplementary file 1 — Additional file 1. Dendrogram of 141 normal samples based on clustering by read depth at all capture regions. Distance between samples was defined as 1 – Pearson correlation of read depth at capture regions. [file 12859_2020_3397_MOESM1_ESM.pdf]
