# Supplementary material for: CNV Radar: an improved method for somatic copy number alteration characterization in oncology
Source: BMC Bioinformatics. 2020 Mar 6;21:98. doi: 10.1186/s12859-020-3397-x (PMC7060549; doi:10.1186/s12859-020-3397-x)
Supplement: Supplementary file 2 — Additional file 2. Supplementary material on running CNVkit, CoNIFER, ExomeDepth and CopywriteR. [file 12859_2020_3397_MOESM2_ESM.pdf]

Additional File 2

Supplementary material

Running CNVKit, CoNIFER, ExomeDepth and CopywriteR

Alignment and post-processing

Post sequencing, Illumina basecalls were converted to FASTQs and aligned to the human genome (HS37d5) using BWA mem 0.7.10. Picard MarkDuplicates (v. 1.47), was used to remove PCR duplicates with validation stringency set to lenient. Read groups were added to the BAM file using Picard AddOrReplaceReadGroups. The Genome Analysis Toolkit (GATK) version 1.6 was further applied to refine the alignments using RealignerTargetCreator and IndelRealigner. The resulting BAM files are used as inputs to CNVkit, CoNIFER, ExomeDepth, and CopywriteR.

CNVKit

CNVKit v8.1 was first executed in access mode (cnvkit.py access) to create an access bed file (-s 10000). Next, CNVKit was run in batch mode (cnvkit.py batch) using all normal, sorted bam files. We specified an exome specific bed file, the access bed file, and used the --split option. After the normal reference file was created, CNVKit was run in batch mode for all samples, using the appropriate reference file to create the cns and cnr files, which were used for all further analysis.

CoNIFER

To run CoNIFER (version 0.2.2), we first calculated the RPKM values for the normal samples. Once these files were available, RPKM values for each tumor sample were calculated, and analyzed alongside the normal RPKM files. CoNIFER allows the user to remove singular value decomposition values. We chose to remove 0, 1, 2, and 3 SVD components respectively and used the Z-RPKM values to assess which regions have CNV event.

ExomeDepth

ExomeDepth (version 1.1.8) was implemented on R (version 3.1.3). It was run by providing a complete list of indexed BAM files, associated with both the tumor and normal samples, for each separate study. Following creation of the counts matrix, CNV calls were made using default parameters and a developer suggested transition probability of 10^{-4} and expected CNV length of 50,000.

CopywriteR

CopywriteR (version 2.12.0) was implemented on R (version 3.5.1). We ran the CopywriteR tool on theTCGA Prostate and AML datasets using the matched tumor/normal samples. MMRF samples, which did not have matched normal tissues, were processed as the tumor only. Segments identified by CopywriteR were then processed by DNAcopy to run circular binary segmentation and determine CNV events using log2 thresholds of -0.25/0.2 for TCGA AML and MMRF, and -0.3/0.3 for TCGA Prostate.

39
