# Supplementary material for: CNV Radar: an improved method for somatic copy number alteration characterization in oncology
Source: BMC Bioinformatics. 2020 Mar 6;21:98. doi: 10.1186/s12859-020-3397-x (PMC7060549; doi:10.1186/s12859-020-3397-x)
Supplement: Supplementary file 3 — Additional file 3. Sensitivity of CNV Radar (blue) and CNVkit (pink). [file 12859_2020_3397_MOESM3_ESM.pdf]

Additional File 3

Sensitivity of CNV Radar (blue) and CNVKit (pink)

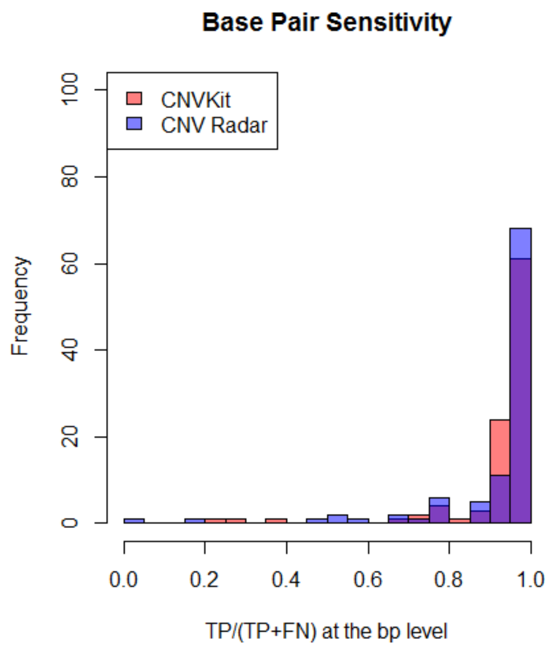

CNV Radar, copy number variation rapid aberration detection and reporting; TP, true positive; FN, false negative.
