# Supplementary material for: CNV Radar: an improved method for somatic copy number alteration characterization in oncology
Source: BMC Bioinformatics. 2020 Mar 6;21:98. doi: 10.1186/s12859-020-3397-x (PMC7060549; doi:10.1186/s12859-020-3397-x)
Supplement: Supplementary file 4 — Additional file 4. Sample SRR2128693 was called more accurately by CNVkit than CNV Radar. Top panel of horizontal bars indicates CNV calls by CNV Radar and CNVkit as well as the true CNV status defined by WGS. [file 12859_2020_3397_MOESM4_ESM.pdf]

Additional File 4

Sample SRR2128693 was called more accurately by CNVKit than CNV Radar. Top panel of horizontal bars indicates CNV calls by CNV Radar and CNVkit as well as the true CNV status defined by WGS.

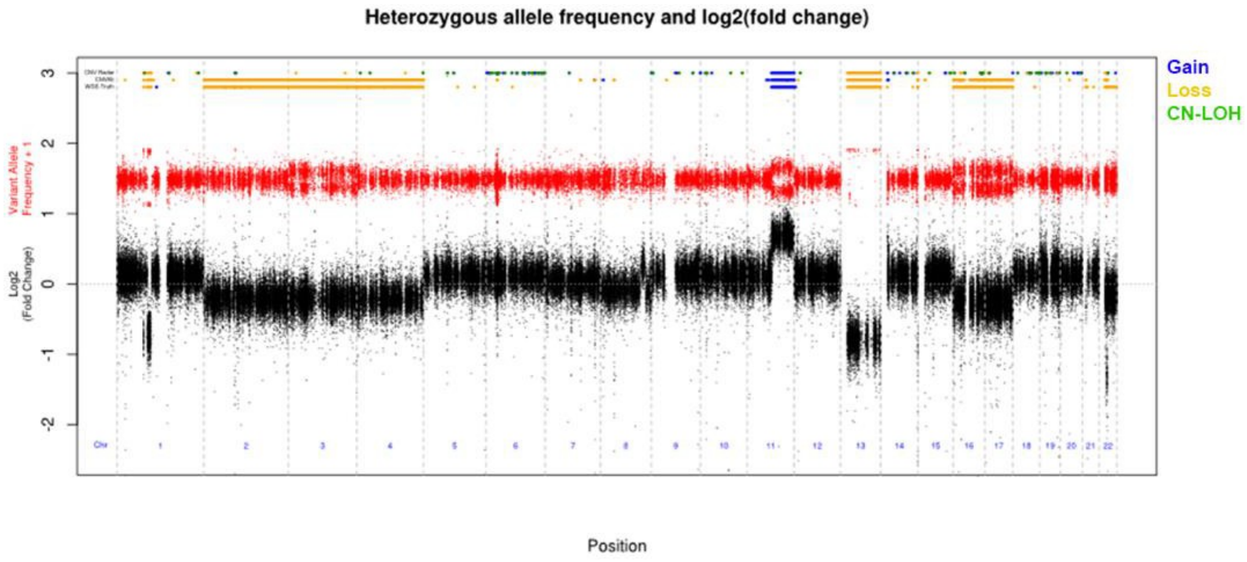

CNV Radar, copy number variation rapid aberration detection and reporting; WGS, whole genome sequencing.
